# Enhancing IoT security through cloud-assisted post-quantum authentication: a case study with UOV signatures

**DOI:** 10.1038/s41598-026-43611-8

**Published:** 2026-04-19

**Authors:** Veeresh R. Maned, Satyabrat Rath, Jothi Ramalingam, Lakshmi Kuppusamy

**Affiliations:** 1 Department of Mathematical and Computational Sciences, National Institute of Technology Karnataka Surathkal, Surathkal, 575025 India; 2 Manipal Institute of Technology, Manipal Academy of Higher Education, Manipal, India; 3 Department of Computer Science and Engineering, Indian Institute of Technology Kharagpur, Kharagpur, India

**Keywords:** Digital signature, Internet of Things, Multivariate cryptography, Post-quantum cryptography, Secure outsourcing, UOV signature scheme, Engineering, Mathematics and computing

## Abstract

The UOV signature scheme is a promising quantum-resistant candidate in NIST PQC, offering shorter signatures and efficiency, but requires solving a system of linear equations, which presents a challenge while performing in resource-constrained IoT devices. This paper introduces the first-ever idea of securely delegating computationally intensive task in UOV signature generation to third-party (cloud) servers, assisting the implementation of post-quantum cryptographic schemes in resource-constrained devices. The proposed “cloud-assisted” UOV signature scheme uses an honest-but-curious cloud framework to facilitate efficient signature generation without compromising security. In addition, this protocol provides a provably secure framework for the privacy of the communicated data (both input and output), for securely generating a UOV signature. The efficacy of the proposed protocol is demonstrated via experimental evaluation and simulations implemented on a Raspberry Pi 5, providing evidence of a reduction in the computational burden and energy consumption in UOV signature generation using our proposed protocol.

## Introduction

This ever-growing generation of the Internet of Things (IoT) introduces exciting possibilities to optimize services and engage with the general population^1^. This framework promotes sustainable development to tackle the growing challenges of urbanization. A critical aspect of IoT is a secure network that connects devices within a digital city, enabling data transmission through wireless technology and cloud systems. IoT devices are integral to smart city architecture, as they collect, analyze, and manage data in real time^2–4^.

The data collected from IoT devices and users is widely shared across the network, making data/user security a critical concern^5–7^. Ensuring secure communication involves verifying the origin of messages, maintaining the integrity of the data during transmission, and protecting it from eavesdroppers. Digital signatures address the concerns of data and entity authentication and data integrity. Most widely deployed digital signature schemes, such as RSA, ECDSA, and others, ensure the security of the signature (or unforgeability), assuming the computational hardness of solving mathematical problems like integer factorization and discrete logarithm.

However, the advent of efficient quantum algorithms poses a serious threat to these schemes. For instance, quantum computers, using Shor’s algorithm^8,9^, can solve the integer factorization problem and discrete logarithm problem in polynomial time, rendering classical schemes like RSA digital signature and ECDSA insecure. Over the past decade, substantial progress in quantum computing has highlighted the need for cryptographic primitives that are resistant to quantum attacks.

In response, the National Institute of Standards and Technology (NIST) initiated the Post-Quantum Cryptography (PQC) Standardization Process in December 2016, which introduced a list of potential digital signature schemes, based on the hard problems of isogeny, algebraic coding theory, lattices, hash functions, and system of multivariate quadratic equations. After four rounds of evaluation, NIST selected two signature schemes based on lattice problems and hash functions for standardization. Additionally, in September 2022, NIST issued a call for more signature schemes, with 4 multivariate signature schemes are still under consideration after Round 2 https://csrc.nist.gov/Projects/pqc-dig-sig/round-2-additional-signatures.

Multivariate signature schemes are designed using a system of multivariate quadratic polynomial equations. Due to its sophisticated design and simple mathematical operations, it presents a tremendous advantage of generating a shorter signature. For instance, at the 128-bit security level, the renowned multivariate signature scheme, Unbalanced Oil and Vinegar (UOV) offers signatures of size 128 bytes, $18.9\times$, and $61.3\times$ shorter than the signature sizes of standardized Crystals-Dilithium (2420 bytes), and SPHINCS$^+$ (7856 bytes) respectively.

However, the concern of generating a faster signature in the UOV signature relies on solving the system of linear equations. The challenge increases when it is implemented in IoT devices, which limits the computational power of the device.

In this paper, we introduce the first-ever idea of securely and efficiently outsourcing the computationally demanding problem of solving a system of linear equations in IoT devices to an honest-but-curious server, while ensuring complete input/output security. This framework enables the IoT devices to generate shorter signatures using UOV, rather than Dilithium and SPHINCS$^+$, without indulging in computationally challenging operations, thus providing a better practical alternative.

### Background

Multivariate public key cryptosystems (MPKCs) form a class of post-quantum cryptographic schemes based on the hardness of solving a system of multivariate quadratic equations over a finite field, an NP-complete problem^10^. Several multivariate signature schemes have been proposed^11–20^, in which the public key consists of multivariate quadratic equations. For systems with more than 16 unknowns, the best-known algorithms remain inefficient, ensuring practical security. To enable feasible signature generation, these schemes employ trapdoor functions, which allow efficient computation when the secret key is known. However, the compromise of certain signature schemes due to structural weaknesses has been well-documented^16,18,21–23^. Nonetheless, many remain secure under rigorous cryptanalysis^15,17,19^.

In 1995, Patarin introduced the Oil and Vinegar (OV) signature scheme^18^, featuring an equal number of oil and vinegar variables. This scheme was later broken by Kipnis and Shamir in 1998 using the invariant subspace attack^21^. In response, the Unbalanced Oil and Vinegar (UOV) signature scheme was proposed in 1999^15^. UOV introduces an asymmetric structure with a larger number of vinegar variables, enhancing its resistance to attacks. Over two decades of cryptanalysis have affirmed the UOV’s resilience, and its exceptional efficiency. The compact signature size make it one of the most promising candidate in the NIST PQC standardization process for security level 1.

### The challenge

UOV signature generation involves solving a system of linear equations, a computationally intensive task for resource-constrained devices. The main challenge in implementing UOV on these devices is their limited memory capacity, restricted battery capacity, and low processing power. These devices lack the computational resources necessary for efficient signature generation, which limits the practical deployment of UOV in such environments^24^.

### The Proposed Approach

To overcome the computational limitations of the resource-constrained devices, securely outsourcing the signature generation to powerful third-party servers offers a promising solution. By delegating computationally expensive tasks to a remote server, devices with limited resources can offload the burden of signature generation while still participating in quantum-safe communication.

However, this outsourcing approach introduces new challenges:

- *Privacy:* The secret key must remain protected during outsourcing.
- *Correctness:* The integrity of the outsourced computation must be verifiable.

This paper presents the first server-aided UOV signature scheme, designed to address these challenges. The proposed protocol ensures efficient and secure signature generation while preserving the core security guarantees of UOV. By enabling resource-constrained devices to leverage powerful servers, the scheme facilitates quantum-safe communication.

## Unbalanced oil and vinegar signature scheme

This section provides an overview of the Unbalanced Oil and Vinegar (UOV) signature scheme, its security assumptions, and the motivation for delegating signature generation in UOV.

A digital signature scheme $\Pi = (KeyGen, Sign, Verify)$ is a tuple of probabilistic polynomial-time algorithms for key generation, signing, and verification.

The UOV signature scheme is defined over a finite field $\mathbb {F}_q$ and consists of two maps:

- $\mathscr {L}: \mathbb {F}_q^{o+v} \rightarrow \mathbb {F}_q^{o+v}$ is an invertible linear transformation.
- $\mathscr {F}: \mathbb {F}_q^{o+v} \rightarrow \mathbb {F}_q^o$ is a central quadratic map comprising *o* multivariate quadratic polynomials $f^{(1)}, f^{(2)},$ $\dots , f^{(o)}$ in $o+v$ variables.

The central map $\mathscr {F}$ is defined as

1 $$\begin{aligned} \mathscr {F}(\mathbf{x}) = \big (f^{(1)}(\mathbf{x}), f^{(2)}(\mathbf{x}), \dots , f^{(o)}(\mathbf{x})\big ), \end{aligned}$$

where each polynomial $f^{(k)}$ for $k = 1, 2, \dots , o$ is given by

2 $$\begin{aligned} f^{(k)}(\mathbf{x}) = \sum _{j=1}^{o+v} \sum _{i=o+1}^{o+v} a_{i,j}^{(k)} x_i x_j, \end{aligned}$$

and $$a_{i,j}^{(k)} \in \mathbb {F}_q$$. Here:

- $x_1, x_2, \dots , x_o$ are the *oil variables*.
- $x_{o+1}, x_{o+2}, \dots , x_{o+v}$ are the *vinegar variables*.

The UOV scheme is termed “unbalanced” as the number of vinegar variables (*v*) exceeds the number of oil variables (*o*). The structure of $\mathscr {F}$ ensures that oil variables do not interact directly in the quadratic polynomials, and the mixing of oil and vinegar variables is obscured by the linear transformation $\mathscr {L}$, which hides the central map structure.

The public key is $\mathscr {P} = \mathscr {F} \circ \mathscr {L}$, and the secret key is $(\mathscr {F}, \mathscr {L})$. The scheme parameters are:

- $o+v$: Total number of variables in the public key polynomials.
- *o*: Number of polynomials in the public key.
- *q*: Field size of $\mathbb {F}_q$.

### Security basis

The security of UOV relies on the difficulty of solving a system of multivariate quadratic equations over $\mathbb {F}_q$, known as the *MQ problem*.

#### Definition 1 (MQ Problem)

Given $o+v, o, q$, and a multivariate quadratic map $\mathscr {P}: \mathbb {F}_q^{o+v} \rightarrow \mathbb {F}_q^o, \mathscr {P}(\textbf{x}) = \big (p^{(1)}(\textbf{x}), p^{(2)}(\textbf{x}), \dots , p^{(o)}(\textbf{x})\big )$, find $\mathbf{w} \in \mathbb {F}_q^{o+v}$ such that:

3 $$\begin{aligned} p^{(1)}(\mathbf{w}) = p^{(2)}(\mathbf{w}) = \cdots = p^{(o)}(\mathbf{w}) = 0. \end{aligned}$$

#### Definition 2 (MQ Assumption)

For every probabilistic polynomial-time algorithm, the probability of finding a solution $\mathbf{w} \in \mathbb {F}_q^{o+v}$ such that $\mathscr {P}(\mathbf{w}) = \mathbf{0}_o$ is negligible.

However, knowledge of $(\mathscr {F}, \mathscr {L})$ enables efficient computation of $\mathscr {P}^{-1}$, as $\mathscr {P}^{-1} = \mathscr {L}^{-1} \circ \mathscr {F}^{-1}$. This trapdoor property underpins the signing algorithm, which is detailed below.

### Algorithms of UOV scheme

The UOV signature scheme comprises the following algorithms:

**Algorithm 1:**
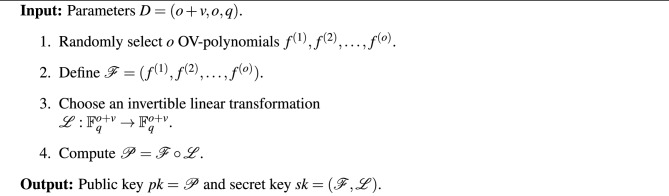
Key generation (*KeyGen*).

**Algorithm 2:**
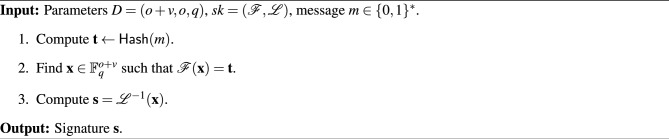
Signing (*Sign*).

**Algorithm 3:**
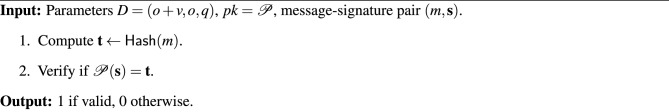
Verification (*Verify*).

### Motivation for delegating signature generation in the UOV scheme

We argue the potential need for offloading the task of signature generation. By outsourcing certain computations, we aim to reduce the computational burden, especially for devices with limited processing power, such as those used in Internet of Things (IoT) applications.

From the discussion in Sect. Unbalanced oil and vinegar signature scheme, the signer in the signature generation algorithm begins by randomly assigning values to the *v* vinegar variables, which simplifies the multivariate quadratic map $\mathscr {F}$ to a more manageable linear map $\Gamma _\mathbf{v}: \mathbb {F}_q^o \rightarrow \mathbb {F}_q^o$. For $q > 2$, this map $\Gamma _\mathbf{v}$ is typically invertible.

The signer then attempts to solve the equation $\Gamma _\mathbf{v}(\mathbf{u}) = \mathbf{t}$ for $\mathbf{u} \in \mathbb {F}_q^o$ using techniques such as Gaussian elimination. If a solution $\mathbf{u}$ is not found, the signer randomly reassigns values to the vinegar variables and repeats the process until a valid solution is obtained. Once $\mathbf{u}$ is determined, the pre-image $\mathbf{x}$ is formed by concatenating $\mathbf{u}$ with the vinegar variables: $\mathbf {x = (u || v)}$.

The process of finding $\mathbf{x}$, particularly solving the linear system of equations $\Gamma _\mathbf{v}(\mathbf{u}) = \mathbf{t}$, can be computationally intensive for resource-constrained devices, as it takes $\mathscr {O}(n^3)$ steps in general. Thus, we propose outsourcing this system-solving task to an external server. By delegating this computationally demanding step in signature generation, the overall computational burden for the signer is significantly reduced. This server-aided approach allows for faster signature generation

## Security model for outsourcing cryptographic computations

We consider a computation framework in which an honest but resource-constrained client $\mathscr {T}$ seeks to outsource an expensive computation task $F: D \rightarrow R$ to a cloud server $\mathscr {U}$. Naturally, $\mathscr {U}$ is not fully trusted by $\mathscr {T}$. Without loss of generality, we say that $(\mathscr {T}, \mathscr {U})$ correctly implements *F* if $F(x) = \mathscr {T}^{\mathscr {U}}(x)$ for all $x \in D$, where $\mathscr {T}^{\mathscr {U}}$ denotes that $\mathscr {U}$ has oracle access to $\mathscr {T}$ and records all its computations over time.

However, the use of an external/remote server $\mathscr {U}$ induces trust issues due to its inaccessibility. Thus, $\mathscr {T}$ may have given access to an untrusted server. There are two main threats when utilizing an untrusted/adversarial server, denoted as $\mathscr {U}'$:

1. A potentially malicious server $\mathscr {U}'$ could attempt to extract confidential information during its interactions with $\mathscr {T}$.
2. $\mathscr {U}'$ may return incorrect results to $\mathscr {T}$’s queries.

Therefore, $\mathscr {T}$ must guarantee:

- *Privacy*: $\mathscr {U}$ cannot gain any useful information of input and output data.
- *Correctness*: $\mathscr {T}$ can verify the correctness of the results returned by $\mathscr {U}$.

Another adversary considered in Ref. 25 is the *environment* $\mathscr {E}$, representing any third party, including the provider or manufacturer of $\mathscr {U}'$. The full adversarial model is represented by the pair $\mathscr {A} = (\mathscr {E}, \mathscr {U}')$. A key assumption in this model is that adversaries $\mathscr {E}$ and $\mathscr {U}'$ can collaborate and plan before $\mathscr {T}$ begins interacting with $\mathscr {U}$. However, once $\mathscr {T}$ starts querying $\mathscr {U}'$, there is no direct communication between $\mathscr {E}$ and $\mathscr {U}'$.

### Security models

Golle and Mironov^26^ introduced the *lazy-but-honest* model for outsourcing the inversion of one-way functions. In this setting, the cloud server $\mathscr {U}$ is viewed as a rational economic agent that attempts to minimize its computational effort while still returning correct results in order to receive payment. Since any incorrect output can be immediately detected by the client $\mathscr {T}$-as verification amounts to recomputing the one-way function–the server is assumed to behave honestly.

Another widely used framework is the *honest-but-curious* (or semi-honest) model, first formalized by Micali *et al.*^27^. Here, all parties strictly follow the prescribed protocol, but one of them-typically $\mathscr {U}$-may attempt to infer additional private information from its protocol view. Thus, while $\mathscr {U}$ produces correct outputs, it may still try to learn sensitive details such as $\mathscr {T}$’s private input or output.

Chen *et al.*^28^ treated the server in both of the above scenarios as a passive adversary. However,there are the situations, where $\mathscr {U}$ may be lazy, curious, and dishonest simultaneously. This strongest threat model is known as the *fully malicious model*^29^. Unlike the *honest-but-curious* model, a server operating under the fully malicious model may return incorrect results for the client’s outsourced computation. Consequently, verification of the computed output is necessary in the fully malicious setting, whereas such verification can be omitted in the *honest-but-curious* model.

Delegation models differ based on the number of servers and/or their adversarial behaviors. We have considered the server in a *honest-but-curious* setup^27,30^ which is most common scenario in IoT environments, where all the IoT devices in the environment are connected to a central node, which interacts honestly with their clients. Its adversarial behavior is captured by $\mathscr {A} = (\mathscr {E},\mathscr {U}')$, where $\mathscr {U}'$ behaves honestly in computation but remains curious about $\mathscr {T}$’s private data.

### Formal definition

Gennaro *et al.*^29^ formalized an *outsourcing computation* scheme as follows.

#### Definition 3

A secure outsourcing computation scheme $\mathsf{OS}$ consists of five polynomial-time algorithms

$$(\mathsf{KeyGen}, \mathsf{ProbGen}, \mathsf{Compute}, \mathsf{Verify}, \mathsf{Solve}):$$

1. $\mathsf{KeyGen}(F,n) \rightarrow (PK, SK)$: *A randomized algorithm that, given a security parameter* *n*, *generates a public key* *PK* *encoding the function* *F* *and a secret key* *SK* *kept by the client* $\mathscr {T}$.
2. $\mathsf{ProbGen}_{SK}(x) \rightarrow (\sigma _x, \tau _x)$: *Using* *SK*, *the client encodes the input* *x* *into a public value* $\sigma _x$ *for the server and a private value* $\tau _x$ *retained locally.*
3. $\mathsf{Compute}_{PK}(\sigma _x) \rightarrow \sigma _y$: *The server computes an encoded output* $\sigma _y$ *corresponding to* $y = F(x)$.
4. $\mathsf{Verify}_{SK}(\sigma _y) \rightarrow \{0,1\}$: *Using* *SK*, *the client checks whether* $\sigma _y$ *is a valid encoding of the correct output.*
5. $\mathsf{Solve}_{SK}(\tau _x, \sigma _y) \rightarrow y$: *The client decodes* $\sigma _y$, *using* $\tau _x$, *to recover the final output* $y = F(x)$.

### Security requirements

A secure outsourcing scheme must guarantee both *correctness* and *security*. Correctness ensures that an honest server produces outputs that always verify and correspond to correct evaluations of *F*. The adversarial setting generally considers the client $\mathscr{T}$ as honest and defines the adversary as $\mathscr {A}=(\mathscr {E},\mathscr {U})$, where $\mathscr {E}$ is an external observer and $\mathscr {U}$ is the server. Modern models typically assume that the server itself behaves adversarially throughout the protocol.

Although the adversary may be computationally powerful, it is always assumed to run in probabilistic polynomial time.

#### Definition 4 (Correctness)

An outsourcing scheme $\mathsf{OS}$ is *correct* if, for any function *F*, whenever $(PK,SK) \leftarrow \mathsf{KeyGen}(F,n)$ and for all $x \in D$, if $(\sigma _x,\tau _x)\leftarrow \mathsf{ProbGen}_{SK}(x)$ and $\sigma _y\leftarrow \mathsf{Compute}_{PK}(\sigma _x)$, then

$$y = F(x) \leftarrow \mathsf{Verify}_{SK}(\tau _x,\sigma _y).$$

Informally, a scheme is *verifiable* if no malicious server can cause the client to accept an incorrect result. In the honest-but-curious server model, correctness always holds.

### Input and output privacy

A robust outsourcing scheme should also protect the client’s privacy. Informally, this means that the encoding generated by $\mathsf{ProbGen}$ must not reveal any information about the plaintext inputs. A scheme provides input privacy if the encoding of two distinct inputs are computationally indistinguishable. This is formalized by the following experiment:

$$\mathsf{Exp}^{\mathsf{Priv}}_{\mathscr {A}}[\mathsf{OS},F,n]:$$

$$\begin{aligned} (PK,SK)&\xleftarrow {\$}\mathsf{KeyGen}(F,n); \\ (x_0,x_1)&\leftarrow {\mathscr {A}}^{\mathsf{ProbGen}_{SK}(\cdot )}; \\ b&\xleftarrow {\$}\{0,1\}; \\ (\sigma _b,\tau _b)&\leftarrow \mathsf{ProbGen}_{SK}(x_b); \\ b'&\leftarrow {\mathscr {A}}^{\mathsf{ProbGen}_{SK}(\cdot )}(PK, x_0, x_1, \sigma _b); \\&\text {If } b' = b, \text { output } 1; \text { else } 0. \end{aligned}$$

#### Definition 5 (Privacy)

For an outsourcing scheme $\mathsf{OS}$, adversary’s advantage in the above experiment is defined as $\textrm{Adv}^{\mathsf{Priv}}_{\mathscr {A}}(\mathsf{OS},F,n) = \Pr [\mathsf{Exp}^{\mathsf{Priv}}_{\mathscr {A}}[\mathsf{OS},F,n] = 1].$ An $\mathsf{OS}$ is *private* if for every probabilistic polynomial-time (*PPT)* adversary $\mathscr {A}$, $\textrm{Adv}^{\mathsf{Priv}}_{\mathscr {A}}(\mathsf{OS},F,n) \le \mathsf{negl}(n)$ for some negligible function $\mathsf{negl}()$.

### Efficiency

An outsourcing scheme must also be efficient: the client should spend significantly less time on encoding the inputs and verifying the outputs than computing *F*(*x*) directly.

#### Definition 6 (Efficiency)

An outsourcing scheme $\mathsf{OS}$ is $\alpha$-efficient if, for all inputs *x*, the computation of *F*(*x*) by $\mathscr {T}$ through a server $\mathscr {U}$ is at least $\alpha$ times faster than computing *F*(*x*) on its own.

## Secure server-aided UOV scheme

In this section, we introduce a server-aided protocol that efficiently generates signatures of the UOV scheme, while ensuring the *outsource-security* of both the secret keys (denoted as $\mathscr {F}$ and $\mathscr {L}$) and the resulting signature $\mathbf{s}$. Unlike existing delegated isogeny-based schemes^31^, our approach requires ONLY a single server and a single round of communication.

Our focus is on providing a secure and viable method to accelerate UOV signature generation in resource-constrained IoT devices. In particular, we aim to offload the computationally intensive task of signature generation in a UOV scheme, enhancing performance without compromising security.

### Precomputation

This section outlines the pre-computation step to restructure the problem of finding the preimage $\mathbf{x} \in \mathbb {F}_{q}^{o+v}$ such that $\mathscr {F}(\mathbf{x}) = \mathbf{t}$, where $\mathbf{t}=(t_1,t_2,...,t_o)=\mathsf{Hash}(m) \in \mathbb {F}_{q}^{o}$ for a given message $m \in \{0,1\}^*$.

By randomly assigning values to the *v* vinegar variables, the signer transforms the map $\mathscr {F}$ (generally, onto) into a linear map $\Gamma _{\mathbf{v}}: \mathbb {F}_q^o \rightarrow \mathbb {F}_q^o$. For this, the signer performs the following steps:

1. *Map Reduction:* The map $\mathscr {F}$ is expressed as in (2)
  $$\begin{aligned} \begin{aligned} \forall k, \ 1 \le k \le o, \ t_k&= f^{(k)}(x_1,x_2,...,x_{o+v}) \\&=\sum _{j=1}^{o+v}\sum _{i=o+1}^{o+v}a_{i,j}^{(k)}x_ix_j. \end{aligned} \end{aligned}$$
2. By selecting the random values from $\mathbb {F}_q$ to the vinegar variables $x_{o+1}, x_{o+2},..., x_{o+v}$, the map $\mathscr {F}$ reduces to $\Gamma _{\mathbf{v}}$ i.e.
  $$\begin{aligned} \sum _{j=1}^{o}\left( \sum _{i=o+1}^{o+v}a_{i,j}^{(k)}x_i\right) x_j+c_k = t_k, \hspace{0.2cm}1\le k \le o, \end{aligned}$$
  where $c_k$ is a constant given by,
  $$\begin{aligned} c_k = \sum _{j=o+1}^{o+v} \sum _{i=o+1}^{o+v}a_{i,j}^{(k)}x_ix_j. \end{aligned}$$
3. *Matrix Representation:* This can be expressed in matrix form as:
  4 $$\begin{aligned} \mathbf{M}\mathbf{u} + \mathbf{c}=\mathbf{t}, \quad \text {or equivalently,} \quad \mathbf{M}\mathbf{u} = \mathbf{t} - \mathbf{c}, \end{aligned}$$
  where $\mathbf{M} \in \mathbb {F}_q^{o \times o}$, $\mathbf{t}=(t_1,t_2,...,t_o), \mathbf{c}=(c_1,c_2,...,c_o),$ $\mathbf{u}=(x_1,x_2,...,x_o) \in \mathbb {F}_q^{o \times 1}. \mathbf{u}$ is an unknown vector to be found, and for $1\le k,j\le o$, the $(k,j)^{th}$ entry of the matrix $\mathbf{M}$ is given by,
  $$\begin{aligned} \mathbf{M}_{kj} = \sum _{i=o+1}^{o+v}a_{i,j}^{(k)}x_{i}. \end{aligned}$$

We propose outsourcing the computation of finding the vector $\mathbf{u}$ in Eq. (4), a task with $\mathscr {O}(o^3 \log q)$ complexity, to facilitate faster signature generation.

### Transformation method

Before detailing the protocol design, we introduce a critical tool for securing the outsourcing process. This tool is a random invertible sparse matrices $\mathbf{Q}_1$ and $\mathbf{Q}_2$ used to obscure the entries of $\mathbf{M}$. It is generated through a secure pseudo-random number generator (PRNG). Throughout our protocol, the term ‘random’ denotes values generated pseudo-randomly by a secure PRNG unless specifically mentioned. The process for generating $\mathbf{Q}_1,\mathbf{Q}_2 \in \mathbb {F}_q^{o \times o}$ is outlined in Algorithm 4.

**Algorithm 4:**
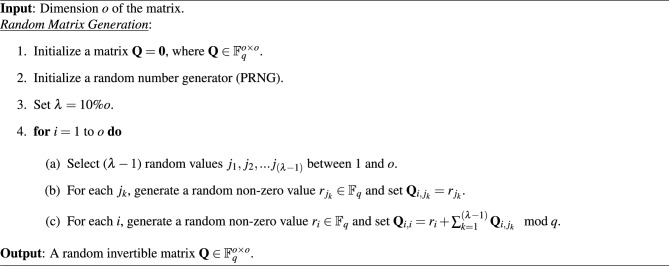
Generate a random invertible sparse matrix $\mathbf{Q}\in \mathbb {F}_q^{o\times o}.$

#### **Transformation of**$\mathbf{M}$

The input coefficient matrix $\mathbf{M}$, which belongs to $\mathbb {F}_q^{o \times o}$, undergoes a transformation by being left and right multiplied by a random sparse matrices $\mathbf{Q}_1$ and $\mathbf{Q}_2$. This multiplication is performed using the field operations defined over $\mathbb {F}_q$. The resulting encrypted matrix is expressed as:

5 $$\begin{aligned} \mathbf{M}_e = \mathbf{Q}_1 \mathbf{M} \mathbf{Q}_2, \quad \mathbf{M}_e \in \mathbb {F}_q^{o \times o}. \end{aligned}$$

Here, $\mathbf{M}_e$ is the transformed version of $\mathbf{M}$, which is of the same dimension as $\mathbf{M}$ but modified to secure its entries during offloading or communication.

#### **Transformation of**$(\mathbf {t-c})$

Similarly, the input vector ($\mathbf {t-c}) \in \mathbb {F}_q^{o \times 1}$ undergoes a two-step transformation. First, it is combined with the vector $\mathbf{Mr} \in \mathbb {F}_q^{o \times 1}$, which is the multiplication of the matrix $\mathbf{M}$ and a random vector $\mathbf{r} \in \mathbb {F}_q^{o \times 1}$. This addition ensures further obfuscation of the data. Subsequently, the result is left-multiplied by the matrix $\mathbf{Q}_1$, which is generated using Algorithm 4, to produce the final encrypted form:

6 $$\begin{aligned} \mathbf {(t - c)}_e = \mathbf{Q}_1(\mathbf {t-c + Mr}), \text {where } \mathbf {(t - c)}_e \in \mathbb {F}_q^{o \times 1}. \end{aligned}$$

#### **Transformation of**$\mathbf{u}$

The output vector $\mathbf{u} \in \mathbb {F}_q^{o \times 1}$ is produced in encrypted form by left multiplying $\mathbf {Q_2}^{-1}$ with the sum of randomly generated vector $\mathbf{r} \in \mathbb {F}_q^{o \times 1}$ to $\mathbf{u}$. This operation uses the standard group addition and matrix multiplication defined over the field $\mathbb {F}_q$. The resultant encrypted vector $\mathbf{u}_e$ is given by:

7 $$\begin{aligned} \mathbf{u}_e = \mathbf{Q}_2^{-1}(\mathbf {u + r}), \quad \mathbf{u}_e \in \mathbb {F}_q^{o \times 1}. \end{aligned}$$

The vector $\mathbf{r}$ acts as a masking element, ensuring that the information in $\mathbf{u}$ is obfuscated while maintaining the same dimensionality.

It can be verified that equations (5), (7), and (6) lead to the relation:

8 $$\begin{aligned} \mathbf{M}_e\mathbf{u}_e = \mathbf {(t - c)}_e. \end{aligned}$$

### The server-aided signing algorithm for UOV

**Fig. 1:**
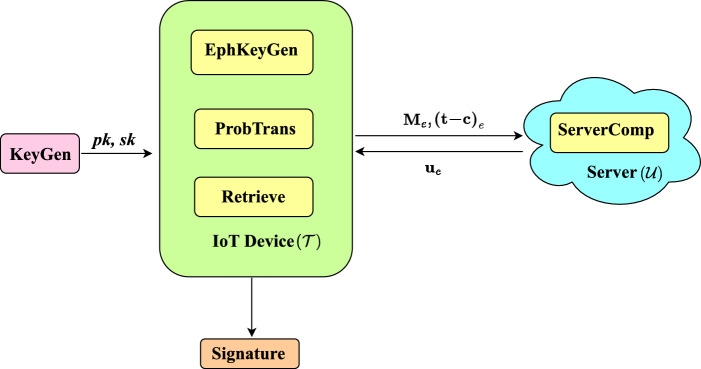
Schematic diagram of the proposed approach.

We now present the server-aided signing algorithm for enabling resource-constrained devices ($\mathscr {T}$) to generate signatures efficiently using an honest-but-curious server $\mathscr {U}$ as shown in the Fig. 1. Our proposed outsourcing protocol $\mathsf{OS}$ is a secret key primitive. It consists of four algorithms: **EphKeyGen**, **ProbTrans**, **ServerComp**, and **Retrieve**.

**Algorithm 5:**
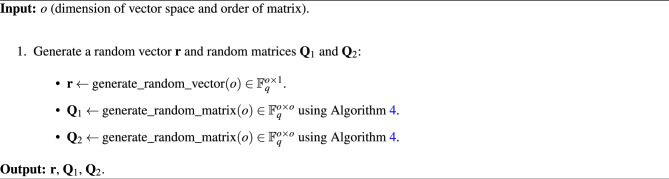
Ephemeral KeyGen algorithm (EphKeyGen).

**Algorithm 6:**
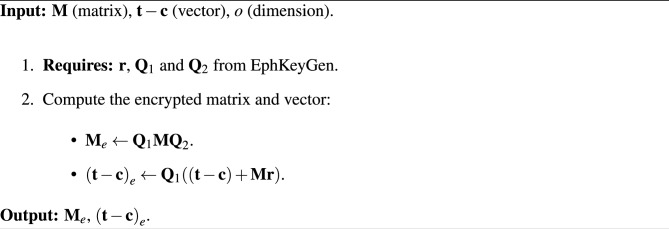
Problem transformation algorithm (ProbTrans).

**Algorithm 7:**
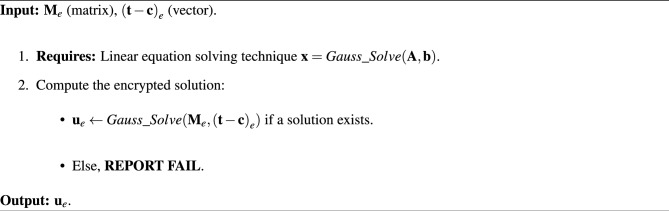
Server computation algorithm (ServerComp).

**Algorithm 8:**
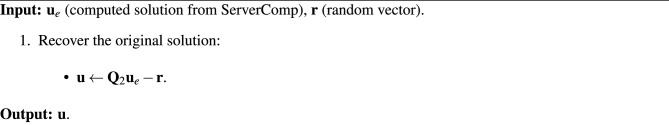
Result retrieval algorithm (Retrieve).

#### Signature generation (Sign)

Once $\mathbf{u}$ is determined, the pre-image $\mathbf{x} \in \mathbb {F}_q^{o+v}$ such that $\mathscr {F}(\mathbf{x}) = \mathbf{t}$, where $\mathbf{t}=\mathsf{Hash}(m) \in \mathbb {F}_q^{o}$ for the message $m \in \{0,1\}^*$, is formed by concatenating $\mathbf{u}$ with the chosen vinegar variables $\mathbf{v}$, i.e.

$$\begin{aligned} \mathbf {x = (u || v)}. \end{aligned}$$

The signature $\mathbf{s}$ is computed as $\mathbf{s} = \mathscr {L}^{-1}(\mathbf{x})$, where $\mathscr {L}^{-1}$ is the inverse of a linear transformation applied to $\mathbf{x}$, generating the signature $\mathbf{s} \in \mathbb {F}_q^{o+v}$.

##### Remark 1

The server is assumed to be honest but curious, i.e. it executes computations correctly but may attempt to learn additional information. As a result, verification of the server’s computed result is not included in $\mathsf{OS}$.

##### Remark 2

The choice of the matrix $\mathbf{Q}$ in this protocol is flexible for the user. Nevertheless, the chosen method to generate $\mathbf{Q}$ provides a security layer for the coefficient matrix $\mathbf{M}$ while ensuring efficiency.

##### Remark 3

There is a possibility that the server may fail to find a solution in Algorithm 7, primarily due to the non-existence of a solution for

$$\begin{aligned} \mathbf{u}_e \leftarrow \text {Gauss\_Solve}(\mathbf{M}_e, \mathbf {(t-c)}_e). \end{aligned}$$

In such instances, the server returns a failure message, indicating that the randomly selected vinegar variables are unsuitable, and the process must be repeated with a new set of random vinegar variables.

## Security and correctness analysis of the outsourcing protocol

We establish the *correctness* and *security* of the proposed outsourcing protocol by evaluating each step in light of the security definitions in Sect. 3.

### Correctness

To demonstrate the correctness of the protocol, we must show that the joint computation performed by the client ($\mathscr {T}$) and the server ($\mathscr {U}$) accurately implements the intended protocol $\mathsf{Alg}$. In this protocol:

- The client generates a random vector $\mathbf{r}$ and the random matrices $\mathbf{Q}_1,\mathbf{Q}_2$ using the **EphKeyGen** algorithm.
- The client encrypts the problem matrix $\mathbf{M}$ and vector ($\mathbf {t - c}$) through the **ProbTrans** algorithm.
- The server computes the solution on the encrypted data using **ServerComp**.
- The client retrieves the final solution using **Retrieve** algorithm.

We must demonstrate that following the steps of the protocol $\mathsf{OS}$, client $\mathscr {T}$ recovers the correct solution $\mathbf{u}$ (Eq. 4) for the original problem.

- *ProbTrans* outputs:
  $$\mathbf{M}_e = \mathbf{Q}_1 \mathbf{M} \mathbf{Q}_2, \quad \mathbf {(t-c)}_e = \mathbf{Q}_1 \left( \mathbf {(t - c) + M r} \right)$$
- The server solves the system
  $$\mathbf{M}_e \mathbf{u}_e = \mathbf {(t - c)}_e.$$
- In *Retrieve*, the client computes:
  $$\mathbf{u} = \mathbf{Q}_2 \mathbf{u}_e - \mathbf{r}$$
  Since
  $$\begin{aligned} \begin{aligned} \mathbf{u}_e&= \mathbf{M}_e^{-1} \cdot \mathbf {(t - c)}_e\\&= (\mathbf{Q}_1 \mathbf{M} \mathbf{Q}_2)^{-1} \mathbf{Q}_1 \mathbf {(t - c + M r)}\\&=\mathbf{Q}_2^{-1} \mathbf{M}^{-1} \mathbf {(t - c + M r)} \end{aligned} \end{aligned}$$
  9 $$\begin{aligned} \begin{aligned} \implies \mathbf{u}&= \mathbf {Q}_2\mathbf {Q}_2^{-1} \mathbf{M}^{-1} \mathbf {(t - c + M r) - r}\\ &= \mathbf{M}^{-1} \mathbf {(t - c) + r - r}\\&= \mathbf{M}^{-1} \mathbf {(t - c)} \end{aligned} \end{aligned}$$
  Thus, the correct solution $\mathbf{u}$ to the original problem $\mathbf {M u = t - c}$ is successfully recovered, proving *correctness*.

### Security analysis

The security of the proposed outsourcing protocol $\mathsf{OS}$ ensures that the inputs and outputs reveal no additional information to adversaries (including external adversaries $\mathscr {E}$ and the untrusted server $\mathscr {U}$) beyond what they are intended to learn from the transcripts of communication.

#### **External adversary**($\mathscr {E}$)

For the security proofs, we consider  *PPT* adversaries, that can perform the computations in a polynomial time. In addition, the (external) adversary is modeled as a passive adversary who can only eavesdrop on the communication between the trusted component $\mathscr {T}$ and the external server $\mathscr {U}$. i.e it just observes $\mathbf{M}_e, \mathbf {(t-c)}_e, \mathbf{u}_e$ and try to learn any meaningful information from these.

In this section, we analyze the security of the proposed outsourcing scheme as per the security requirements defined in Sect. Security requirements.

**Attack game 1** ($\mathbf{M}_e, \mathbf {(t-c)}_e$**Distinguisher**)

**Fig. 2:**
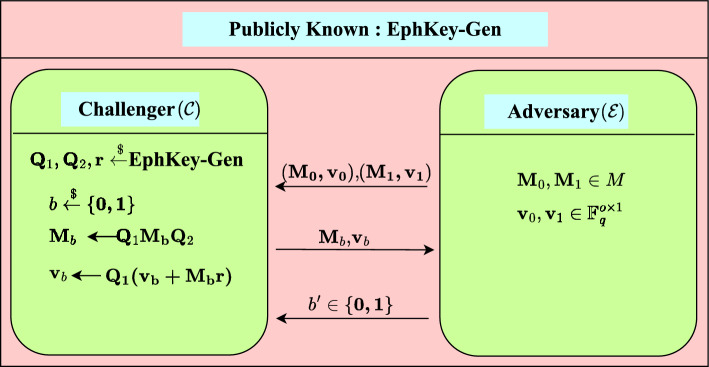
Attack Game 1 : $\mathbf{M}_e, \mathbf {(t-c)}_e$ Distinguisher.

First, we discuss the adversary’s advantage in distinguishing between the two non-singular, non-scalar matrices and non-zero vector masked as per the algorithm 6, employing truly random sparse matrices and a vector using the protocol as detailed in 4.3 through an attack game as shown in the Fig. 2:

It consists of a challenger $\mathscr {C}$ and an adversary $\mathscr {E}$. The algorithm **EphKey-Gen** for generating the masking matrices $\mathbf{Q}_1, \mathbf{Q}_2$ and a vector **r** is made public. Let *K* denote the collection of matrices generated using the algorithm 5, *M* denote the collection of $o \times o$ non-singular, non-scalar matrices over $\mathbb {F}_q$, and *C* denote the collection of matrices of the form $\mathbf{Q}_1 \mathbf{M} \mathbf{Q}_2$ for $\mathbf{Q}_1, \mathbf{Q}_2 \in$ *K*, and $\mathbf{M} \in$ *M*.

1. Challenger $\mathscr {C}$ generates truly random matrices $\mathbf{Q}_1, \mathbf{Q}_2$ and a vector $\mathbf{r}$ using the algorithm 5, and keeps it with itself.
2. Adversary $\mathscr {E}$ chooses two non-singular, non-scalar matrices $\mathbf{M}_0,\mathbf{M}_1 \in M$, and the vectors $\mathbf{v}_0, \mathbf{v}_1 \in \mathbb {F}_q^{o\times 1}$, then sends the pair ($\mathbf{M}_0,\mathbf{v}_0$) and ($\mathbf{M}_1,\mathbf{v}_1$) to $\mathscr {C}$.
3. $\mathscr {C}$ randomly selects a bit *b* from $\{0,1\}$ and computes $\mathbf{M}_{b_e} \leftarrow \mathbf{Q}_1\mathbf{M}_b \mathbf{Q}_2, \mathbf{v}_{b_e} \leftarrow \mathbf{Q}_1(\mathbf{v}_b+\mathbf{M}_{b}\mathbf{r})$. Then $\textbf{M}_{b_e}$, $\textbf{v}_{b_e}$ are sent to $\mathscr {E}$.
4. $$\mathscr {E}$$ outputs a bit $$b' \in \{0,1\}$$.

$\mathscr {E}$ wins the attack game if $b'= b$. Let $\mathscr {D}\_adv[\mathscr {E}, n]$ denote the probability of adversary $\mathscr {E}$ winning in the Attack game 1.

Since $|K| < |M|$, it is not possible to achieve perfect indistinguishability. Then there exist a pair of matrices $\textbf{M}_0,\textbf{M}_1 \in M$ such that for an arbitrary $\textbf{C} \in C$, $\text {Pr}_{\textbf{Q}_1,\textbf{Q}_2 \leftarrow K}[\textbf{Q}_1\textbf{M}_0\textbf{Q}_2 = \textbf{C}] \ne$ $\text {Pr}_{\textbf{Q}_1,\textbf{Q}_2 \leftarrow K}[\textbf{Q}_1\textbf{M}_1\textbf{Q}_2 = \textbf{C}]$. The adversary may attempt to find two such matrices $\textbf{M}_0,\textbf{M}_1$ using the method described below.

Choose $\textbf{M}_0 \in M$ arbitrarily, then compute $\textbf{C}_0= \textbf{Q}_1\textbf{M}_0\textbf{Q}_2$ for some $\textbf{Q}_1, \textbf{Q}_2$ generated using the Algorithm 5. Then, $\text {Pr}_{\textbf{Q}_1,\textbf{Q}_2 \leftarrow K}[\textbf{Q}_1\textbf{M}_0\textbf{Q}_2 = \textbf{C}_0] \ne 0$. Construct the set $S = \{\textbf{Q}_{1}^{-1} \textbf{C}_0 \textbf{Q}_{2}^{-1}: \textbf{Q}_1,\textbf{Q}_2 \in K\}$. Since $|K|<|M|$, $S \subset M$. Next select $\textbf{M}_1 \in M \setminus S$ that guarantees $\text {Pr}_{\textbf{Q}_1,\textbf{Q}_2 \leftarrow K}[\textbf{Q}_1\textbf{M}_1\textbf{Q}_2 = \textbf{C}_0] = 0$. Now, the adversary $\mathscr {E}$ can distinguish $\textbf{M}_0$ from $\textbf{M}_1$ with significant probability if it receives the masked matrix as $\mathbf {C_0}$. $\mathscr {C}$ outputs $\mathbf {C_0}$ when the Algortihm 5 generates exactly the same $\textbf{Q}_1,\textbf{Q}_2$ that $\mathscr {A}$ used to construct the set *S*. The probability of this event is $$\frac{1}{|K|^2}=\frac{1}{\left( \left( {\begin{array}{c}o\\ \lambda -1\end{array}}\right) (q-1)^{\lambda }\right) ^{2o}}$$, which is negligible for the values of *o*, and *q* recommended in (https://www.uovsig.org/).

When challenger outputs $\mathbf {C \ne C}_0$, a *PPT* adversary may try polynomial number of times in demasking $\mathbf{C}$, with the success probability of each attempt being $$\frac{1}{\left( \left( {\begin{array}{c}o\\ \lambda -1\end{array}}\right) (q-1)^{\lambda }\right) ^{o}}$$ resulting in the total probability of $$\frac{\mathsf{poly}(n)}{\left( \left( {\begin{array}{c}o\\ \lambda -1\end{array}}\right) (q-1)^{\lambda }\right) ^{o}}$$, which is again negligible.

Since $\mathbf{v}_b$ is masked by adding $\mathbf{M}_b \mathbf{r}$ for a random vector $\mathbf{r} \in \mathbb {F}_q^{o\times 1}$, it achieves perfect indistinguishability when considered independent of $\mathbf{M}_b$. Nevertheless, under analysis with $\mathbf{M}_b$, the adversary’s ability to distinguish is no less than its distinguishing advantage for $\mathbf{M}_b$. Hence, we have

10 $$\begin{aligned} \begin{aligned} \mathscr {D}\_adv[\mathscr {E}, o]&=Pr[\mathscr {E} \hspace{0.05cm} outputs \hspace{0.05cm}b': b'=b ]\\&\le \frac{1}{2}+\text {max}\left\{ \frac{1}{\left( \left( {\begin{array}{c}o\\ \lambda -1\end{array}}\right) (q-1)^{\lambda }\right) ^{2o}},\frac{\mathsf{poly}(n)}{\left( \left( {\begin{array}{c}o\\ \lambda -1\end{array}}\right) (q-1)^{\lambda }\right) ^{o}}\right\} \\&\le \frac{1}{2}+\frac{\mathsf{poly}(n)}{\left( \left( {\begin{array}{c}o\\ \lambda -1\end{array}}\right) (q-1)^{\lambda }\right) ^{o}}. \end{aligned} \end{aligned}$$

Therefore, for every *PPT* adversary, matrix and vector masking of the proposed outsourcing protocol is computationally indistinguishable.

**Attack game 2 (Input Privacy)**

To analyse the input privacy of the proposed outsourcing protocol, we discuss the *PPT* adversary’s advantage in distinguishing the transcripts of real-world and ideal world simulations. We consider two games $\mathbf{G}_0$ and $\mathbf{G}_1$ corresponding to real world and ideal world simulations detailed as below:

**Game** $\mathbf{G}_0:$ It consists of a challeneger $\mathscr {C}$ and an adversary $\mathscr {A}$ and the game is played as described in the attack game 1 (External adversary$\mathscr {E}$ ) above.

**Game** $\mathbf{G}_1:$ This game is similar to $\mathbf{G}_0$ except the response of the challenger to the queries ($\mathbf{M}_0,\mathbf{v}_0$), ($\mathbf{M}_1,\mathbf{v}_1$). Challenger randomly outputs $(\mathbf{R,t}) \in \mathbb{G}\mathbb{L}(o,\mathbb {F}_q) \times \mathbb {F}_q^{o\times 1}$.

**Fig. 3:**
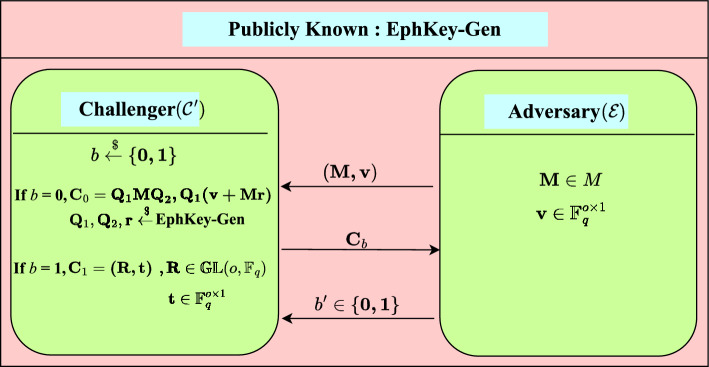
Attack Game 2: Input Privacy.

Now consider the attack game between challenger $\mathscr {C}'$ and an adversary $\mathscr {E}$ which is played as shown in Fig. 3.

1. Adversary $\mathscr {E}$ chooses a non-singular, non-scalar matrix from *M* and a non-zero vector $\mathbf{v} \in \mathbb {F}_q^{o\times 1}$ and sends it to $\mathscr {C}'$.
2. $\mathscr {C}'$ selects a random bit *b* from $\{0,1\}$. It computes $\mathbf{C}_b$ accordingly.
  $$\begin{aligned} \mathbf{C}_b = {\left\{ \begin{array}{ll} (\mathbf{Q}_1\mathbf{M}\mathbf{Q}_2, \mathbf{Q}_1(\mathbf {v+Mr})) \hspace{0.2cm} \text {where} \hspace{0.2cm}\mathbf{Q}_1, \mathbf{Q}_2, \mathbf{r} \leftarrow \mathbf{EphKeyGen}(n), & \text {if } b = 0 \\ \\ (\mathbf{R,t}), & \text {if } b = 1 \end{array}\right. } \end{aligned}$$
  Then $\mathbf{C}_b$ is sent to $\mathscr {E}$.
3. $$\mathscr {E}$$ outputs $$b' \in \{0,1\}$$.

$\mathscr {E}$ wins the attack game if $b'= b$. Let $\mathbf{In}\_adv[\mathscr {E}, \mathbf {EphKey-Gen}]$ denote the probability of adversary $\mathscr {E}$ winning in this game. $b = 0$, outputs the transcript of game $\mathbf{G}_0$, while $b = 1$, that of the game $\mathbf{G}_1$. Output of the challenge phase in $\mathbf{G}_0$ is $\mathbf{M}_e, \mathbf {(t-c)}_e$, whereas that of $\mathbf{G}_1$ is a random matrix, vector pair ($\mathbf{R,t}$) from $\mathbb{G}\mathbb{L}(o,\mathbb {F}_q) \times \mathbb {F}_q^{o\times 1}$. For a given $\mathbf{M}$, distributions of $\mathbf{Mr}$ and $\mathbf{r}$ are same, when $\mathbf{r}$ is uniformly distributed over $\mathbb {F}_q^{o\times 1}$. Hence, $\mathbf{Q}_1(\mathbf {v+Mr})$ and $\textbf{t}$ are perfectly indistinguishable. So, the adversary’s advantage in distinguishing the outputs of challenge phase of $\mathbf{G}_0$ and $\mathbf{G}_1$ depends on the distribution of $\mathbf{M}_e$. For an arbitrary $\mathbf{C} \in \mathbb{G}\mathbb{L}(o,\mathbb {F}_q)$ the probability that there exist $\mathbf{Q}_1, \mathbf{Q}_2 \in K$ such that $\mathbf{Q}_1\mathbf{M}\mathbf{Q}_2=\mathbf{C}$ is $$\frac{\left( \left( {\begin{array}{c}o\\ \lambda -1\end{array}}\right) (q-1)^{\lambda }\right) ^{o}}{\left( \left( {\begin{array}{c}o\\ \lambda -1\end{array}}\right) (q-1)^{\lambda }\right) ^{2o}}$$. Thus, winning the **Input Privacy** game reduces to distinguishing the distribution of $\mathbf{M}_e$ from the uniform distribution over $\mathbb{G}\mathbb{L}(o,\mathbb {F}_q)$. i.e.

11 $$\begin{aligned} \begin{aligned} \mathbf{In}\_adv[\mathscr {E}, \mathbf {EphKey-Gen}]&=Pr[\mathscr {E} \hspace{0.05cm} outputs \hspace{0.05cm}b': b'=b ]\\&= |Pr[\mathscr {E} \text {outputs \textit{b}}'=1|b=0]-Pr[\mathscr {E} \text {outputs \textit{b}}'=1|b=1]|\\&= \left| \frac{\left( \left( {\begin{array}{c}o\\ \lambda -1\end{array}}\right) (q-1)^{\lambda }\right) ^{o}}{\left( \left( {\begin{array}{c}o\\ \lambda -1\end{array}}\right) (q-1)^{\lambda }\right) ^{2o}} - \frac{1}{|\mathbb{G}\mathbb{L}(o,\mathbb {F}_q)|}\right| \\&= \left| \frac{1}{\left( \left( {\begin{array}{c}o\\ \lambda -1\end{array}}\right) (q-1)^{\lambda }\right) ^{o}} - \frac{1}{|\mathbb{G}\mathbb{L}(o,\mathbb {F}_q)|}\right| \\&< \frac{1}{\left( \left( {\begin{array}{c}o\\ \lambda -1\end{array}}\right) (q-1)^{\lambda }\right) ^{o}}. \end{aligned} \end{aligned}$$

For the values of *o*, and *q* recommended in (https://www.uovsig.org/) $$\frac{1}{\left( \left( {\begin{array}{c}o\\ \lambda -1\end{array}}\right) (q-1)^{\lambda }\right) ^{o}}$$ is negligible.Therefore, games $\mathbf{G}_0$ and $\mathbf{G}_1$ are computationally indistinguishable.

**Attack game 3 (Output Privacy)**

It is clear from the equation 7, that the output vector $\mathbf{u} \in \mathbb {F}_q^{o \times 1}$ of the proposed outsourcing protocol is produced in obfuscated form by adding a random vector $\mathbf{r} \in \mathbb {F}_q^{o \times 1}$ to $\mathbf{u}$. Therefore, $\mathbf{u}_e$ is perfectly indistinguishable from a random vector chosen uniformly from $\mathbb {F}_q^{o \times 1}$.

From the attack games 1, 2, and 3 we have shown that, the real and ideal views of an external adversary are computationally indistinguishable:

$$\mathscr {E}\textsf{VIEW}_{\text {real}} \sim \mathscr {E}\textsf{VIEW}_{\text {ideal}}.$$

#### **Untrusted server**($\mathscr {U}$)

The external server, to which the task of solving a system of linear equations has been delegated, is assumed to be honest but curious. While it performs the computations accurately, it may also be interested in uncovering the contents of the encrypted data it receives. Similar security arguments can be applied to analyze $\mathscr {U}$’s ability to infer $\mathbf{M}$, $\mathbf{t}$, $\mathbf{c}$, and $\mathbf{u}$ from $\mathbf{M}_e$, $\mathbf {(t-c)}_e$, and $\mathbf{u}_e$, as in the security analysis for $\mathscr {E}$.

In the ideal world, the server $\mathscr {U}$ learns nothing beyond the fact that it is solving a system of linear equations. The simulator $S_2$ can produce an indistinguishable view by generating random matrices and vectors without needing access to the original inputs. Consequently, the real and ideal views for the server are computationally indistinguishable:

$$\mathscr {U}\textsf{VIEW}_{\text {real}} \sim \mathscr {U}\textsf{VIEW}_{\text {ideal}}.$$

#### **Security of the proposed outsourcing protocol**

In the proposed outsourcing protocol, we use a secure pseudorandom generator (PRNG) (*G*) to generate the matrix $\mathbf{Q}$ in the Algorithm 4. Let

$$G:\{0,1\}^{s}\rightarrow \{0,1\}^{\ell }$$

be a cryptographically secure PRNG that expands an $s$-bit seed into an $\ell$-bit output, with $\ell \gg s$. The output of $G$ is used to deterministically generate an invertible sparse matrix $\mathbf{Q}\in \mathbb {F}_q^{o\times o}$ via Algorithm 4, as described below.

The PRNG output is interpreted as a binary stream and parsed sequentially to derive the structure and entries of $\mathbf{Q}$.

1. The $\ell$-bit output of $G$ is treated as a bit stream and partitioned into substrings of appropriate lengths as required during the construction.
2. To consruct an $o\times o$ sparse matrix with exactly $\lambda$ nonzero entries per row over $\mathbb {F}_q$, at least
  $$o\big ((\lambda -1)\log _2 o + \lambda \log _2 q\big )$$
  bits are required in expectation. However, additional bits may be consumed due to the rejection-sampling procedure described below.
3. For each row, the positions of the $\lambda$ nonzero entries are selected by parsing successive blocks of length $\log _2 o$. Each block is interpreted as an integer in $\{0,\ldots ,o-1\}$. If a block evaluates to zero, it is discarded and the next $\log _2 o$-bit block is considered. This process continues until $(\lambda -1)$ valid nonzero positions are obtained.
4. Once the positions of the nonzero entries in a row are fixed, their corresponding values are generated by parsing the remaining bit string from the previous step. In particular, $\lambda$ blocks of length $\log _2 q$ are extracted with each block interpreted as an element of $\mathbb {F}_q$. Blocks that evaluate to zero are discarded and replaced by subsequent $\log _2 q$-bit block, thereby ensuring that all selected entries are nonzero elements of the field.

Finally, a random vector $\mathbf{r}\in \mathbb {F}_q^{o\times 1}$ is derived by parsing the remaining PRNG output into $o$ consecutive blocks of length $\log _2 q$, each interpreted as an element of $\mathbb {F}_q$.

Next, we show that, under the assumption $\textsf{VIEW}_{\text {real}} \sim \textsf{VIEW}_{\text {ideal}}$, the matrix and vector masking in the proposed outsourcing protocol 4.3 using a secure PRNG is computationally indistinguishable through the following attack game.

##### Proposition 1

Let $G: \{0,1\}^s \rightarrow \{0,1\}^\ell$ be a PRNG, and $\mathsf{OS}= \{{\mathbf {EphKeyGen}}, {\mathbf {ProbTrans}}, {\mathbf {ServerComp}}, {\mathbf {Retrieve}}\}$ be the outsourcing protocol. If *G* is a secure PRNG, then the matrix and vector masking of $\mathsf{OS}$ is computationally indistinguishable when $\textsf{VIEW}_{\text {real}} \sim \textsf{VIEW}_{\text {ideal}}$.

##### Proof

**Fig. 4:**
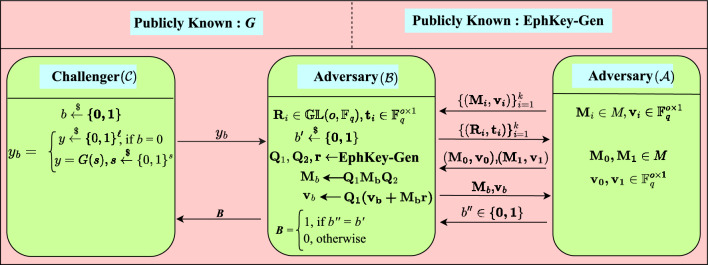
Attack game 4: security of outsourcing protocol.

Proof is established through an attack game (**Attack game 4**) as shown in Fig. 4. It consists of an adversary $\mathscr {A}$ against the another adversary $\mathscr {B}$ in the $\mathbf{M}_e, \mathbf {(t-c)}_e$ distinguishing game. $\mathscr {B}$ acts as the challenger for $\mathscr {A}$. There exists a challenger $\mathscr {C}$ for the adversary $\mathscr {B}$ in the PRNG distinguishing game. The assumption $\textsf{VIEW}_{\text {real}} \sim \textsf{VIEW}_{\text {ideal}}$ is made for the adversary $\mathscr {A}$. Through reduction we will show that, if $\mathscr {A}$ distinguishes the matrix and vector masking with non-negligible probability, then this adversary can be run as the subroutine by the adversary $\mathscr {B}$ to distinguish PRNG from a truly random string with non-negligible probability. The game proceeds as follows: $\square$

1. Challenger $\mathscr {C}$ randomly chooses a bit *b* from $\{0, 1\}$. A $\ell$- bit string $y_b$ is computed as
  $$\begin{aligned} y_b = {\left\{ \begin{array}{ll} y \xleftarrow {\$}\{0,1\}^\ell , & \text {if } b = 0 \\ \\ y = G(s) \hspace{0.1cm} \text {for }s\xleftarrow {\$}\{0,1\}^s, & \text {if } b = 1 \end{array}\right. } \end{aligned}$$
  Then it is forwarded to $\mathscr {B}$.
2. If the adversary $\mathscr {A}$ queries $\mathscr {B}$ with ($\mathbf{M}_i,\mathbf{v}_i$) for masking ($i = 1,2,\cdots k$ ), it outputs ($\mathbf{R}_i, \mathbf{t}_i$) by randomly choosing $\mathbf{R}_i$ from $\mathbb{G}\mathbb{L}(o, \mathbb {F}_q)$ and $\mathbf{t}_i$ from $\mathbb {F}_q^{o \times 1}$. The assumption $\textsf{VIEW}_{\text {real}} \sim \textsf{VIEW}_{\text {ideal}}$ gives the view for $\mathscr {A}$ as in the real-world masking.
3. In challenge phase $\mathscr {A}$ sends the pair ($\mathbf{M}_0,\mathbf{v}_0$) and ($\mathbf{M}_1,\mathbf{v}_1$) to $\mathscr {B}$, where $\mathbf{M}_0,\mathbf{M}_1 \in M$ are the non-singular, non-scalar matrices, and the vectors $\mathbf{v}_0, \mathbf{v}_1 \in \mathbb {F}_q^{o\times 1}$.
4. $\mathscr {B}$ parses the binary string $y_b$ as detailed above to construct the matrices $\mathbf {Q_1, Q_2}$ and the vector $\textbf{r}$. Then it randomly selects a bit $b'$ from $\{0,1\}$, and computes $\mathbf{M}_{b_e} \leftarrow \mathbf{Q}_1\mathbf{M}_b \mathbf{Q}_2, \mathbf{v}_{b_e} \leftarrow \mathbf{Q}_1(\mathbf{v}_b+\mathbf{M}_{b}\mathbf{r})$. It sends $\mathbf{M}_{b_e}$, $\mathbf{v}_{b_e}$ to $\mathscr {A}$.
5. $$\mathscr {A}$$ outputs a bit $$b'' \in \{0,1\}$$.
6. The adversary $\mathscr {B}$ outputs the bit *B* as
  $$\begin{aligned} B = {\left\{ \begin{array}{ll} 1, & \text {if } b'' = b' \\ \\ 0, & \text {otherwise } \end{array}\right. } \end{aligned}$$.

$\mathscr {A}$ wins if $b'' = b'$. Let $\textbf{M}\_adv[\mathscr {A}, \mathbf {EphKey-Gen}]$, and $\mathbf{PRNG}\_adv[\mathscr {B}, G]$ denote the distinguishing advantages of the adversaries $\mathscr {A}$ and $\mathscr {B}$ respectively. Now, consider the distinguishing advantage of $\mathscr {B}$.

12 $$\begin{aligned} \mathbf{PRNG}\_adv[\mathscr {B}, G] = |Pr[ \mathscr {B} \hspace{0.1cm}\text {outputs } B=1|b=0]- Pr[ \mathscr {B} \hspace{0.1cm}\text {outputs } B=1|b=1] |. \end{aligned}$$

$$Pr[ \mathscr {B} \hspace{0.1cm}\text {outputs } B=1|b=0] = Pr[ \mathscr {A} \hspace{0.1cm}\text {outputs } b''=b' \text { when a truly random string is used}]$$

$$Pr[ \mathscr {B} \hspace{0.1cm}\text {outputs } B=1|b=1] = Pr[ \mathscr {A} \hspace{0.1cm}\text {outputs } b''=b' \text { when PRNG is used}]$$.

Since *G* is a secure PRNG, $\textbf{PRNG}\_adv[\mathscr {B}, G] \le \mathsf{negl}(n)$ for some negligible function $\mathsf {negl()}$, and from, Eq. (10) we have $$Pr[ \mathscr {B} \hspace{0.1cm}\text {outputs } B=1|b=0] \le \frac{1}{2}+\frac{\mathsf{poly}(n)}{\left( \left( {\begin{array}{c}o\\ \lambda -1\end{array}}\right) (q-1)^{\lambda }\right) ^{o}}$$. This implies,

13 $$\begin{aligned} Pr[ \mathscr {A} \hspace{0.1cm}\text {outputs } b''=b' \text { when PRNG is used}] \le \frac{1}{2}+\frac{\mathsf{poly}(n)}{\left( \left( {\begin{array}{c}o\\ \lambda -1\end{array}}\right) (q-1)^{\lambda }\right) ^{o}} + \mathsf{negl}(n). \end{aligned}$$

This establishes, that the matrix and vector masking in the proposed outsourcing protocol 4.3 using a secure PRNG is computationally indistinguishable.

#### **Security of server-aided UOV scheme**

In the following section, we are going to establish that, the security of the server-aided UOV scheme follows from the security of the UOV scheme through the following result.

##### Proposition 2

Let $\Pi = (KeyGen,Sign,Verify)$, and $\Pi _s = ({KeyGen}_s, {Sign}_s, {Verify}_s)$ denote the UOV, and server-aided UOV signature scheme respectively. If $\Pi$ is a secure UOV signature scheme, then $\Pi _s$ is a secure server-aided UOV signature scheme when the real-world and ideal-world executions of the server-aided signature generation are computationally indistinguishable.

##### Proof

This proof is formalized using an attack game (*Attack game 5*). Considering all the assumptions in the statement we will deduce that if there is an adversary that produces a valid forgery in the server-aided UOV signature scheme, then this adversary can be used to produce a valid forgery in the UOV signature scheme, which contradicts the assumption in the statement. $\square$

**Fig. 5:**
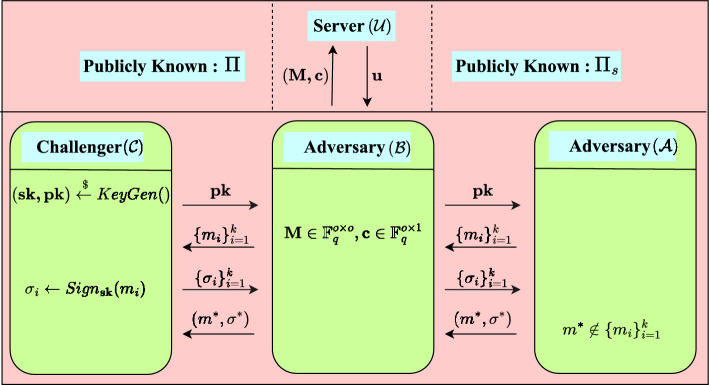
Attack game 5: security of server-aided UOV scheme.

It consists of an adversary $\mathscr {A}$, which outputs a valid forgery in the server-aided UOV signature scheme, an adversary $\mathscr {B}$ which uses the forgery of $\mathscr {A}$ to produce a valid forgery in the UOV signature scheme, an external server $\mathscr {U}$, and the challenger $\mathscr {C}$ for the adversary $\mathscr {B}$. Here $\mathscr {B}$ acts like a challenger for $\mathscr {A}$. The assumption $\mathscr {A}\textsf{VIEW}_{\text {real}} \sim \mathscr {A}\textsf{VIEW}_{\text {ideal}}$ is made for the adversary $\mathscr {A}$. The attack game shown in the Fig. 5 is played as described below:

1. To begin $\mathscr {C}$ runs the key generation algorithm *KeyGen* to get the key pair $(\mathbf{sk,pk})$. Secret key$(\mathbf{sk})$ $=(\mathscr {L},\mathscr {F})$, Public key$(\mathbf{pk})$ $=\mathscr {P}$. $\mathscr {C}$ sends $\mathscr {P}$ to $\mathscr {B}$, which it forwards to $\mathscr {A}$.
2. $\mathscr {A}$ queries for signatures on the polynomial number of messages $m_1,m_2,...,m_k$ to $\mathscr {B}$. $\mathscr {B}$ forwards the same to $\mathscr {C}$. To give the view of server-aided UOV signature generation for $\mathscr {A}$, $\mathscr {B}$ randomly generates a matrix $\mathbf{M}\in \mathbb {F}_q^{o \times o}$, a vector $\mathbf{c}\in \mathbb {F}_q^{o \times 1}$, and sends $\mathbf{M,c}$ to the server $\mathscr {U}$ for solving the system of linear equations. The assumption $\mathscr {A}\textsf{VIEW}_{\text {real}} \sim \mathscr {A}\textsf{VIEW}_{\text {ideal}}$ gives $\mathscr {A}$ the view of server-aided UOV signature generation.
3. $\mathscr {C}$ computes the signatures $\sigma _i \leftarrow Sign(m_i)$ for $i=1,2,...,k$ and sends the signatures $\sigma _1,\sigma _2,...,\sigma _k$ to $\mathscr {B}$ corresponding to the messages $m_1,m_2,...,m_k$. $\mathscr {B}$ forwards the signatures $\sigma _i$ on messages $m_i$ as if it generated these signatures in the server-aided UOV signature generation setting.
4. Then $\mathscr {A}$ replies with a forgery ($m^*, \sigma ^*$) to $\mathscr {B}$ such that $m^* \notin \{m_1,m_2,...,m_k\}$, and it forwards the same to $\mathscr {C}$.

Let $Forg\_adv[\mathscr {B}, \Pi ]$, and $Forg\_adv[\mathscr {A}, \Pi _s]$ denote the forgery probabilities of the adversaries $\mathscr {B}$, and $\mathscr {A}$ respectively. In the above attack game, the view of $\mathscr {A}$ when run as a subroutine by $\mathscr {B}$ is distributed exactly as its view in the attack game with its challenger. Since $\mathscr {B}$ outputs a valid forgery when $\mathscr {A}$ generates, we have

14 $$\begin{aligned} Forg\_adv[\mathscr {A}, \Pi _s] = Forg\_adv[\mathscr {B}, \Pi ]. \end{aligned}$$

By the security assumption of $\Pi$, $Forg\_adv[\mathscr {B}, \Pi ]$ is negligible. Consequently, the server-aided UOV scheme $\Pi _s$ is a secure signature scheme.

### Efficiency and checkability analysis

#### **Efficiency**

This section presents a rigorous theoretical analysis of the efficiency of our proposed outsourcing protocol. Specifically, we compare the computational cost of our protocol with that of the original UOV scheme to demonstrate its computational efficiency. *o*, and *v* denote the number of oil and vinegar variables respectively.

**Table 1:** Theoretical efficiency analysis of the proposed protocol.

Protocol algorithms	Time complexity
Ephemeral key generation (*EphKeyGen*)	$\mathscr {O}(o)$
Problem transformation (*ProbTrans*)	$\mathscr {O}(o^2)$
Result retrieval (*Retrieve*)	$\mathscr {O}(o)$
Signature generation (*Sign*)	$\mathscr {O}((o+v)^2)$
Communication rounds	1

Table 1 summarizes the computational complexities associated with each phase of our protocol.

- **EphKeyGen**: The cost of generating the random matrix $\mathbf{Q}$ is $\mathscr {O}(o)$ (using the Algorithm 4).
- **ProbTrans**: This phase involves two matrix-matrix multiplications $\mathbf{Q}_1 \mathbf{M}$, $(\mathbf{Q}_1 \mathbf{M})\mathbf{Q}_2$ and two matrix-vector multiplications, leading to a computational complexity of $\mathscr {O}(o^2)$.
- **Retrieve**: Retrieving the original solution from the encrypted form, as described in Algorithm 8, involves a matrix-vector multiplication and vector subtraction, resulting in computational complexity of $\mathscr {O}(o)$.
- **Sign**: This phase involves finding $\mathscr {L}^{-1}(\mathbf {x)}$. It is matrix-vector multiplication (since every linear transformation can be uniquely represented as a matrix), with a complexity of $\mathscr {O}((o+v)^2)$.

##### Remark 4

In the Problem Transformation algorithm, **ProbTrans**, it’s important to note that, as each row of $\mathbf{Q}_1,\mathbf{Q}_2$ contains only $\lambda$ non-zero entries, ($\lambda = 10\% \text { of }o$), the total number of multiplications in $\mathbf{Q}_1\mathbf{M}\mathbf{Q}_2$ is $2\lambda o^2$. Therefore, time complexity of matrix-matrix multiplication ($\mathbf{Q}_1\mathbf{M}\mathbf{Q}_2$) is $\mathscr {O}(o^2)$ instead of $\mathscr {O}(o^3)$.

##### Proposition 3

The proposed outsourcing protocol $\mathsf{OS}$ achieves *o*-efficiency for an honest-but-curious server $\mathscr {U}$.

##### Proof

The signature generation of the original UOV scheme requires $\mathscr {O}(o^3 + (o+v)^2)$ time. As demonstrated in the efficiency analysis, the proposed protocol reduces this complexity to $\mathscr {O}(o^2 + (o+v)^2)$. Hence, the efficiency factor is

$$\alpha = \frac{o^3 + (o+v)^2}{o^2 + (o+v)^2} \approx \frac{o^3}{o^2} = o.$$

Therefore, the proposed protocol is *o*-efficient.

##### Remark 5

If the external server is malicious, the client will verify the server’s result by checking whether $\mathbf {Mu\overset{?}{=}\ t}$. This involves a matrix-vector multiplication with time complexity $\mathscr {O}(o^2)$, which does not increase the overall time complexity of the Signature Generation (**Sign**). Since $\mathbf{Q}_1\mathbf{M}\mathbf{Q}_2$ is an invertible matrix, the equation

$$\begin{aligned} \mathbf{M}_e \mathbf{u}_e = \mathbf {(t - c)}_e \end{aligned}$$

has a unique solution. Hence, the correctness of the solution returned by a potentially malicious server can be verified.

## Comparison with existing outsourcing cryptographic schemes

Table 2 presents a comparison of representative works on secure outsourcing of cryptographic computations. The comparison is carried out with respect to the number of servers involved, the number of communication rounds, the outsourced cryptographic task, and the assumed threat model. Most prior works primarily focus on the secure outsourcing of modular exponentiation, a core operation in many classical public-key cryptosystems. In 2021, Pedersen^31^ was the first to propose secure delegation of isogeny computations in the CSIDH, a post-quantum cryptosystem. More recently, in 2024, Ramalingam et al.^32^ introduced a scheme for secure outsourcing of information reconciliation in quantum key distribution (QKD). In contrast, our work is the first to propose a server-aided UOV signature generation protocol, requiring only a single round of communication with an honest-but-curious server.

**Table 2:** Comparison of secure delegation of expensive cryptographic computations.

Work	Number of rounds	Outsourced cryptographic task	Number of servers	Threat model
Micali, Goldreich, & Wigderson^27^ (1987)	Multi	Multi-part computations	> 2	Honest but curious
Susan, Anna^25^ (2005)	1	Modular exponentiation in Cramer- Shoup encryption & Schnorr signatures	2	Malicious
Chevallier-Mames et al.^33^ (2010)	1	Elliptic curve pairing	1	Honest but Curious
Gennaro, Gentry, Parno^29^ (2010)	1	Garbled circuit evaluation (in the context of FHE^34^)	1	Malicious
Chen et al.^35^ (2013)	Multi	Modular exponentiation in Cramer- Shoup encryption & Schnorr signatures	2	Malicious
Backes et al.^36^ (2013)	Multi	Quadratic polynomials computation with large number of variables	1	Malicious
Kiraz et al.^37^ (2015)	1	Modular exponentiation in Oblivious Transfer Protocols & Blind Signatures	1	Malicious
Chevalier, Laguillaumie, & Vergnaud^30^ (2021)	1	Modular exponentiation	1	Honest but Curious
R Pedersen^31^ (2021)	2	Isogeny computation in CSIDH	2	Malicious, Honest but Curious
Rath, Ramalingam, Lee^38^ (2024)	1	Modular exponentiation in RSA	1	Malicious
Ramalingam et al.^32^ (2024)	1	Information Reconciliation in QKD	1	Malicious
Our work	1	Solving system of linear equations in UOV signature generation	1	Honest but Curious

## Experimental analysis

This section outlines the experimental setup and results that validate the theoretical claims presented in this work. The inputs (values of *o*, *v*, and *q*) taken for the experiments are considered as mentioned in the NIST documentation (https://www.uovsig.org/).

The client-side computations ($\mathscr {T}$) were performed on Raspberry Pi 5. The server-side computations ($\mathscr {U}$) were executed on Linux 20.04, 11th Gen IntelCore i7-11700 @ 2.5GHz octa-core processor, 16 GB RAM, and 512 GB SSD. (The entire implementation was developed in a Jupyter environment using Python version 3.13.5.) Raspberry Pi and Linux were connected using a WiFi network with a network band of 2.4 GHz/5 GHz and link speed of 1 Mbps.

**Table 3:** End-to-end time consumption (in milliseconds) for various steps in Server-aided UOV signature generation.

Sl. no.	*o*	*v*	*q*	EphKeyGen +ProbTrans	Retrieve	Server-aided signing time
1	44	68	256	5.1	0.5	5.6
2	64	96	16	7.4	0.5	8.1
3	72	112	256	10.2	0.7	10.9
4	96	148	256	13.7	0.8	14.5

**Fig. 6:**
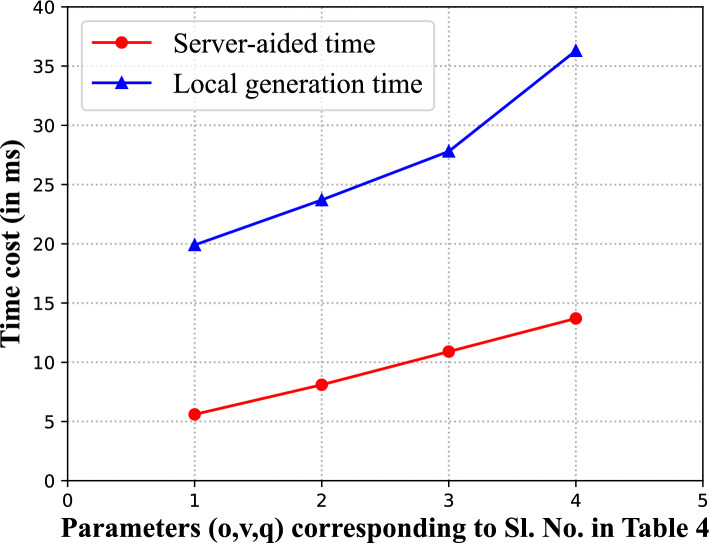
Runtime comparison (in milliseconds) of UOV signature generation: server-aided Signature generation vs. Local signature generation (unaided).

**Table 4:** Runtime comparison (in milliseconds) of UOV signature generation: Server-aided signing vs. Local signing (unaided).

Sl. No.	*o*	*v*	*q*	Server-aided signing	Local signing time
1	44	68	256	5.6	19.9
2	64	96	16	8.1	23.7
3	72	112	256	10.9	27.8
4	96	148	256	14.5	36.3

**Table 5:** Energy consumption (in milli Joules): Server-aided signing v/s Local signing.

Sl.	*o*	*v*	*q*	Server-aided signing (mJ)	Local signing (mJ)
1	44	68	256	24.6	90.2
2	64	96	16	39.7	118.5
3	72	112	256	57.8	161.2
4	96	148	256	82.6	214.1

**Table 6:** Communication overhead (in KB).

Sl.	*o*	*v*	*q*	Client to server	Server to client	Total
1	44	68	256	1.94	0.044	1.984
2	64	96	16	2.08	0.032	2.112
3	72	112	256	5.256	0.072	5.328
4	96	148	256	9.312	0.096	9.408

For the experimental study, we followed the parameters $o< v < o^2$ as suggested in Ref. 15. We compared the runtime performance of signature generation between the standard UOV scheme and the proposed outsourcing protocol. To obtain reliable performance metrics, we averaged the computation time across 20 instances, keeping the number of oil (*o*) and vinegar (*v*) variables constant. The end-to-end time consumption for various steps in server aided UOV signature generation is presented in Table 3. It includes the phases of **Ephemeral Key Generation**, **Problem Transformation**, **Server-side Computation**, **Retrieval** (The *Retrieve* phase in our experimental setup also encompasses the computation required for the Final Signature Generation), and**Server-aided signing time.** The Raspberry Pi communicates with the server using an ssh-based client–server protocol. In this setup, the authentication latency was measured to be approximately 200–240 ms.

Comparative runtime results for UOV signature generation are presented in Table 4 and plotted in Fig. 6. The time required for generating a UOV signature locally on the user’s device, without external assistance, is referred to as the **Local signing time**, whereas the total time incurred when employing the proposed server-aided UOV signature generation protocol is denoted as the **Server-aided signing**. As observed from Tables 3 and 4, the server-aided approach reduces the computational burden on the signer by approximately $\sim$ 2.5 - 3.5 $\times$. It is worth noting that, in the server-aided setting, the overall computation time is influenced by network latency and available bandwidth; in our experimental setup, the authentication phase required approximately 200–240 ms for a WiFi network.

**Fig. 7:**
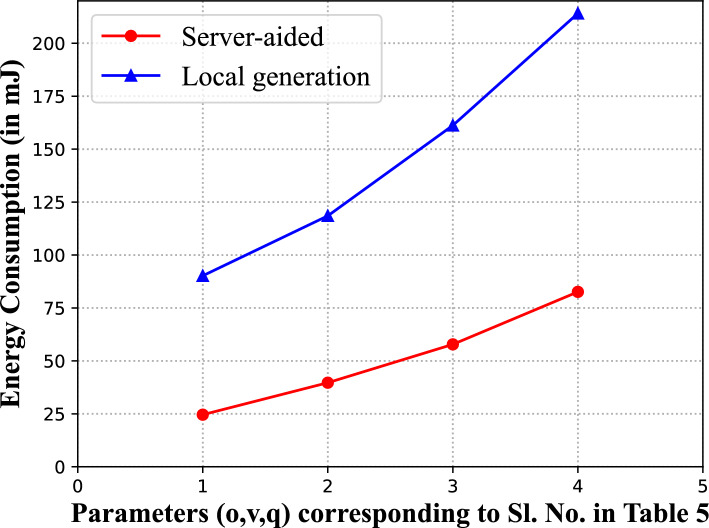
Energy consumption comparison (in millijoules) of UOV signature generation: server-aided signing vs. local signing.

Comparative energy consumption results for signature generation are reported in Table 5 and plotted in Fig. 7. The results indicate that server-aided signing consumes less energy than local signature generation. Since resource-constrained devices are typically battery-powered, the proposed protocol can potentially extend device operational lifetime. The communication overhead of the protocol is summarized in Table 6. The observed communication cost is below 10 KB, which appears to be feasible for deployment in resource-constrained environments.

Our simulations confirmed that the proposed outsourcing protocol produces signatures identical to those generated by the original UOV signature scheme. Despite the impact of network latency on the overall server-aided computation time, the proposed outsourcing protocol substantially reduces the computational load and energy consumption at the client compared to local signature generation. As a result, the energy savings achieved through the server-aided approach can extend the operational lifetime of battery-powered, resource-constrained devices. The results clearly indicate the efficiency and practicality of outsourcing the computational workload to the cloud.

## Conclusion

We have revisited the UOV scheme, which has successfully withstood cryptanalysis for over two decades. However, the computational demands of UOV signature generation often prevent resource-limited devices from participating in quantum-safe communication, limiting the broader adoption of UOV.

To address this, we proposed a server-aided UOV signature scheme, the first of its kind, enabling resource-constrained devices to efficiently generate UOV signatures without compromising security. The efficacy of our proposed protocol is demonstrated through its implementation on Raspberry Pi 5. The performance evaluation, energy consumption tables clearly highlight its efficiency and practicality. Our approach is generic, which can be applied to variants of UOV such as Salted UOV, PRUOV (Provable UOV), TUOV (Triangular UOV), SNOVA, QR-UOV, and MAYO. The last three are also currently under consideration by NIST for additional post-quantum signature schemes.

## Data Availability

No datasets were generated or analysed during the current study.
